# Clinical Decision Support for Digital Dietary Counseling Under GLP-1 Receptor Agonist Therapy: Convergent Mixed Methods Usability and Treatment Satisfaction Pilot Study of the Personalized Nutrition Advisor

**DOI:** 10.2196/81112

**Published:** 2026-07-21

**Authors:** Freya Orban, Arlette Journeaux, Philipp Kanehl, Melanie Stoll, Marco Lehmann, Felix Schirmann, David Herzig, Lia Bally

**Affiliations:** 1 Department of Diabetes, Endocrinology, Nutritional Medicine and Metabolism, Faculty of Medicine, University of Bern, Freiburgstrasse 15, Bern, 3010, Switzerland, +41 31 632 40 70; 2 Graduate School for Cellular and Biomedical Sciences, Bern University, Bern, Switzerland; 3 Oviva AG, Berlin, Germany

**Keywords:** usability, mHealth, decision support system, digital nutrition, weight management, weight loss, overweight

## Abstract

**Background:**

Dietary counseling is an essential complement to the growing use of glucagon-like peptide-1 receptor agonists (GLP-1 RAs) to achieve sustainable weight loss. Diverse patient needs, the demand for ongoing support, and limited resources underscore the potential of using digital support tools.

**Objective:**

This study aimed to evaluate the usability, treatment satisfaction, and weight effects of a personalized nutrition advisor (PNA), a prototype decision-support tool designed to facilitate personalized weight management counseling.

**Methods:**

We assessed usability, treatment satisfaction, and weight effects of a web-based PNA prototype dashboard designed to help dietitians deliver personalized remote dietary counseling in overweight adults (BMI ≥28 kg/m² at pharmacotherapy initiation) who had achieved ≥5% weight loss with GLP-1 RA therapy over 12-weeks. The PNA dashboard generated tailored nutritional recommendations to help participants achieve personalized weight loss goals based on an energy balance model that estimated daily caloric intake and nutritional quality using frequent body weight measurements, dietary logs, and physical activity data from a lifestyle-tracking app. To evaluate the PNA’s usability, we used a convergent parallel mixed-methods approach, combining a validated quantitative usability survey and qualitative focus-group interviews and feedback questionnaires with both dietitians and patients, alongside patient-reported satisfaction metrics for PNA-guided consultations (treatment satisfaction, general self-efficacy, and perceived health impact). Thematic analysis with a hybrid deductive-inductive methodology was applied to qualitative data, using 4 a priori themes derived from this study’s conceptual framework regarding prior expectations, perceived utility and satisfaction, usability challenges, and suggestions for clinical integration. Percent weight loss during the intervention was assessed as an exploratory outcome.

**Results:**

Among 78 participants (58/78, 74% women), on GLP-1 RA (mean weight loss of −13.3%, SD 6.7%, after 10, SD 5, months of treatment), between 3 and 5 remote nutrition consultations were delivered with PNA support. After the 3-month intervention period, weight loss was sustained in all participants (−0.2%, SD 2.5%), with 21% (16/78) suspending the GLP-1 RA due to drug shortages. Dietitians rated the PNA’s usability as moderate (62%) on the Healthcare Systems Usability Scale. Patients evaluated the perceived health impact of the PNA-assisted consultations at 19.1 (SD 3.5) of 25 on the user version of the Mobile Application Rating Scale. No significant differences were observed in patient-reported treatment satisfaction (*P*=.78) or self-efficacy (*P*=.57) before and after PNA-assisted care. Qualitative analysis identified convergence with quantitative findings across themes of usability, treatment satisfaction, and clinical integration, with divergence observed for self-efficacy and tracking motivation. Both patients and dietitians found the PNA valuable for generating actionable nutritional insights.

**Conclusions:**

While effective implementation of tools such as the PNA prototype demands dietitian training and workflow integration, they offer scalable, personalized nutrition or lifestyle guidance—as a therapeutic decision-support tool within weight loss interventions, with or without adjunct GLP-1 RA pharmacotherapy.

## Introduction

Dietitian-led counseling remains the gold standard for personalized dietary care in obesity management, a role that becomes even more critical with the growing use of glucagon-like peptide-1 (GLP-1) pharmacotherapy. However, access to dietitian services is constrained by workforce capacity, reimbursement structures, and the inherent limitations of episodic consultations with limited time to review personal data [1,2].

Digital health tools offer a complementary pathway to address these gaps [3]. Mobile health apps and wearable technologies enable continuous self-tracking of diet, physical activity, and body weight, generating rich data that can be harnessed through advanced data analytics to tailor dietary recommendations [4]. Of particular interest are weight prediction models embedded within these apps that enable personalized, data-driven insights and tailored advice for achieving predefined weight goals. The energy balance model developed by Hall [5] provides the theoretical and computational basis for predicting weight changes in response to lifestyle interventions, offering essential insights for personalized treatment optimization. For example, by enhancing an energy balance model with Bayesian inference, continuous data gathered from a lifestyle tracking app can infer dietary patterns beyond energy intake estimations [6,7], building the foundation for personalized nutritional advice.

The use of digital tools to support lifestyle optimization through personalized nutritional advice is of growing interest for managing metabolic diseases such as diabetes and obesity. For example, a recently published randomized controlled trial demonstrated the clinical efficacy of a digital, AI-driven Diabetes Prevention Program in adults with prediabetes and overweight or obesity [8]. However, we are not aware of any evaluated personalized nutrition advisor (PNA) tool that combines body weight prediction via an energy balance model with data from a lifestyle tracking app in the context of GLP-1-based weight management.

Several nutritional priorities arise in the context of GLP-1-based weight management [9]. Another growing concern is that weight regain following glucagon-like peptide-1 receptor agonist (GLP-1 RA) discontinuation is substantial and rapid [10,11]. Energy restriction due to loss of appetite can predispose individuals to nutritional deficiencies (eg, micronutrients) or inadequate protein intake, with deleterious effects on lean mass and bone mineral density [12-15]. Furthermore, GLP-1 RA–related side effects may require specific dietary measures to increase drug tolerability (eg, avoidance of fatty and spicy foods or carbonated beverages). When weight stabilizes, or when drug tapering or discontinuation is planned, specific calorie goals become important to maintain body weight and avoid regain. Energy balance models help to set these goals.

This work aimed to evaluate the usability, treatment satisfaction, and weight effects of a PNA tool that embeds an energy balance model and leverages data from a lifestyle tracking app to deliver tailored recommendations and support dietitians’ remote nutritional counseling. Clinical usability, treatment satisfaction, and weight effect outcomes were assessed in a single-group mixed methods study in which the tool was used by dietitians to support nutritional counseling of adults during the weight maintenance phase after achieving clinically significant weight loss using GLP-1 RA therapy.

## Methods

### Study Design and Population

This study used a prospective single-arm convergent parallel mixed methods design [16] in which quantitative and qualitative data were collected simultaneously, and with equal priority, analyzed independently, and subsequently integrated at the interpretation stage to obtain a more complete understanding of PNA usability and treatment satisfaction than either method could provide alone. This design was selected because evaluating the usability and treatment satisfaction of a novel prototype requires both the breadth of standardized quantitative measurement and the depth of qualitative exploration to capture the contextual and experiential dimensions of clinical implementation that validated scales alone cannot adequately reflect [17,18].

This trial enrolled overweight adults (BMI ≥28 kg/m² before GLP-1 RA initialization) who had achieved ≥5% weight loss with GLP-1 RA to support ongoing weight management over 12 consecutive weeks of PNA-assisted remote nutritional counseling using the Oviva lifestyle-tracking app [19]. Remote nutritional counseling was delivered by 6 certified dietitians representing the full complement of practitioners within the program.

### Recruitment and Sampling

Participants were consecutively referred via the dietitians from the outpatient weight specialist clinic at the University Hospital Bern between August 2023 and May 2024. Inclusion criteria were being aged ≥18 years; having a minimum of 16 weeks of GLP-1 RA therapy in combination with remote nutritional counseling and use of the lifestyle tracking app, ensuring participants had passed the initial rapid, predominantly drug-induced weight loss phase; and achieved a clinically significant weight loss of ≥5% (for baseline BMI 28‐35 kg/m²) or ≥7% (for baseline BMI ≥35 kg/m²) at the time of enrollment. The 16-week minimum also ensured that sufficient historical data from the lifestyle-tracking app were available to inform the PNA tool’s energy balance model from day 1 of the intervention. Exclusion criteria were the inability to provide written informed consent; any physical or psychological condition likely to interfere with study conduct or interpretation of results; self-reported pregnancy, planned pregnancy within 3 months, or breastfeeding; concomitant participation in another interfering trial; and insufficient proficiency in German (as this study’s related questionnaires were only available in German).

Of 105 individuals screened for eligibility, 81 met the inclusion criteria and were enrolled. The sample size was calculated for the primary patient outcome (treatment satisfaction), assuming a proportion of *P*=.20, a margin of error of 0.1, and a 95% confidence level (z=1.96), yielding a required sample size of 62 completed questionnaires. Accounting for an anticipated dropout rate of 20%, a total of 80 participants were planned for recruitment.

### Ethical Considerations

This study was approved by the Ethics Committee of Bern (2023‐00864) and registered at ClinicalTrials.gov (NCT05997771). All participants provided written informed consent before any study-related procedures. All data were deidentified, and no participant compensation was provided.

### PNA

The PNA is a prototype clinical decision-support tool designed to visually present patients’ current dietary patterns, energy balance, and body weight trends to enhance nutritional counseling and deliver actionable, individualized recommendations aligned with each patient’s weight goals and nutritional needs. The PNA integrates 3 primary data sources: frequent body weight measurements, logged food intake, and physical activity data from a lifestyle-tracking app. These inputs feed into a mathematical model that forecasts weight trajectories by estimating energy intake and expenditure [7].

**Figure 1.**
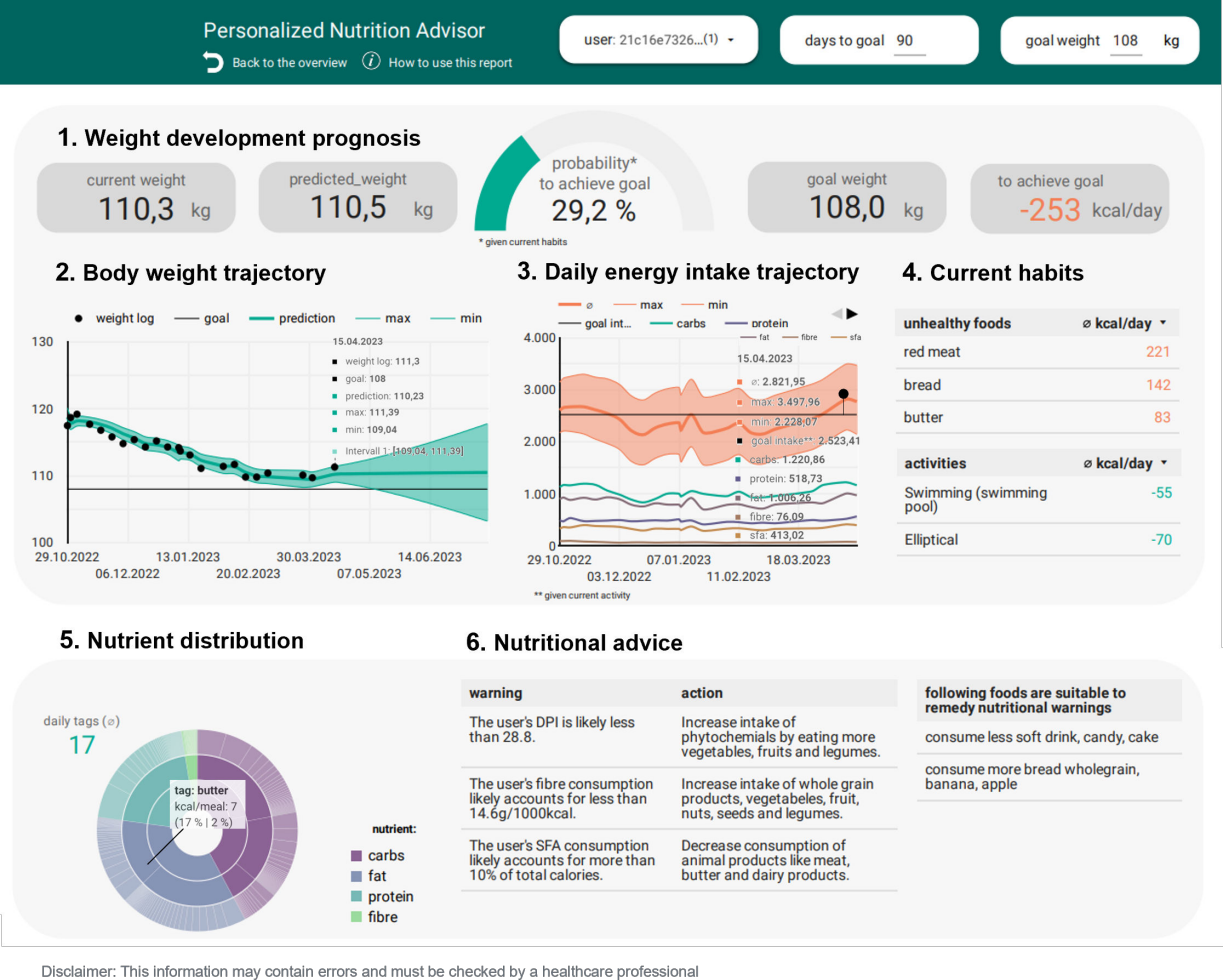
Interactive web-based PNA dashboard for supporting nutritional counseling under GLP-1 RA therapy. The dashboard displays six interactive panels: (1) weight development, (2) body weight trajectory, (3) daily energy intake trajectory, (4) current habits, (5) nutrient distribution, and (6) nutritional advice. DPI: dietary phytochemical index; GLP-1 RA: glucagon-like peptide-1 receptor agonist; PNA: personalized nutrition advisor; SFA: saturated fatty acids.

To facilitate interactive use, the PNA prototype was implemented as an interactive web-based dashboard via Google Looker Studio, visualizing model outputs through six integrated panels (Figure 1):

1. Weight development prognosis: shows the patient’s current weight, predicted weight at the target date, probability of achieving the target weight, and the deviation from the estimated daily energy intake required to reach the goal within the specified timeframe.
2. Body weight trajectory: visualizes all retrospective weight logs alongside the projected weight trajectory and its uncertainty range (high-density interval), enabling users to compare actual and predicted progress.
3. Daily energy intake trajectory: displays estimated daily total energy intake with uncertainty ranges, as well as estimated caloric intake per macronutrient group, providing insights into overall energy intake patterns.
4. Current habits: summarizes the daily caloric impact on energy intake and expenditure of the most frequently tagged foods and activities over the past month, highlighting high-energy foods and activity-energy correlations.
5. Nutrient distribution: presents a pie chart of total and relative daily caloric intake by macronutrient and by individually tracked foods (food tags), and shows the mean number of daily food tags logged in recent months as an indicator of dietary data completeness.
6. Nutritional advice: highlights excesses or deficits in nutritional components compared to a complete diet plan (Table S1 in Multimedia Appendix 1), providing all essential nutrients. It provides actionable dietary recommendations based on each user’s tracked foods. Warnings and recommendations are generated when observed data indicate at least a 90% probability of deviating from diet plan thresholds based on the Swiss dietary recommendations from the Federal Food Safety and Veterinary Office [20].

To support realistic and individualized weight goal setting, the PNA enables adjustment of the output estimation timeframe via the dashboard’s upper control bars. As a PDF printout of the PNA output could be distributed to patients via email by their dietitian, a clear disclaimer indicating that the information is to be reviewed by a health care professional only and must not replace professional medical or dietary advice was included.

The dietary data outputs and visualization components of the PNA were developed through an iterative expert review process involving senior registered dietitians with dedicated prototype development roles and without involvement in the prospective usability evaluation. Feedback was systematically incorporated across multiple development cycles to ensure the clinical plausibility and interpretability of the tool’s dietary pattern assessments and recommendations. Formal criterion validation against an established dietary assessment reference method was not conducted.

One month before this trial’s period, all dietitians received comprehensive training on this trial’s purpose and objectives, including a 1-hour hands-on demonstration of PNA system navigation and functionality delivered by the research team. Following the training, all dietitians had access to the PNA dashboard to get used to it before the intervention started. To support ongoing reference and skill development, dietitians were provided with multiple supplementary training resources, including a 20-minute video tutorial of how to use the dashboard, a comprehensive procedure manual, and a condensed 1-page reference guide. No formal competency assessment was conducted following training, but dietitians were encouraged to consult the research team for technical queries or other PNA-related questions arising before the intervention started but not during this trial to avoid outcome interference by the research team.

### PNA-Assisted Remote Nutritional Counseling

The PNA prototype was evaluated within the established remote nutritional counseling framework provided by Oviva, which consists of a professional platform enabling certified dietitians to access insights from their patients’ app-based lifestyle-tracking (eg, weight, nutrition, and activity logs) and provide feedback through various communication channels (video calls, phone calls, and secure messaging).

The lifestyle-tracking app for patients enables dietary intake tracking using photographs or text entry, physical activity monitoring (entered manually or synchronized via connected devices such as Apple Health, Google Fit, or Fitbit [Google LLC]), and body weight measurements (entered manually or via connected TRANSTEK Teleweight [Guangdong Transtek Medical Electronics Co, Ltd] scales) [19,21]. Food items in photographs were automatically recognized and assigned 1 of 117 predefined food tags by a proprietary deep-learning image recognition algorithm that has been quality validated previously [22]. Automatically assigned food tags could be discarded, confirmed, or modified by users. Only user-confirmed tags were incorporated into the PNA model. Participants were encouraged to track body weight at least once weekly using connected scales or manual entry.

To access the PNA dashboard, dietitians were required to use an access-restricted link to Google Looker Studio. They were encouraged to access the dashboard during both the preparation and delivery of each nutritional counseling session. Dietitians had the option to share the PNA report with patients in PDF format via email when they determined that patients would benefit from having access to their personalized nutritional insights. Dietitians were asked to track their time of PNA usage (including time used for both preparation and consultation) for each patient within a study-specific document. To preserve real-world conditions, dietitians worked at their typical pace without additional time accommodations to discuss or share the PNA outputs.

### Patient Characteristics, Lifestyle Behavior, and Weight Trajectories

Patient sociodemographic and socioeconomic characteristics were collected at study initiation via an online questionnaire. Medical history, comorbidities, and anthropometric measurements, including body weight at the commencement of GLP-1 RA therapy, along with treatment-specific information, were extracted from electronic medical records. All available body weight measurements, dietary intake logs, and physical activity data (both pre and during the PNA trial period) were automatically extracted from the lifestyle-tracking app and used for the weight prediction model and descriptive analysis of app-engagement. Descriptive information regarding remote counseling sessions during this study’s period was retrieved from the digital platform used by the dietitians.

### Quantitative Usability Assessment—Dietitians Delivering PNA-Assisted Consultations

Using the validated Healthcare Systems Usability Scale (HSUS), dietitians assessed the PNA’s usability upon study completion (online). Items 2 and 22 were excluded as they were not applicable to this study’s scope. Each item was evaluated using a 5-point Likert scale (1-5), where higher scores indicate greater agreement. We calculated the mean score, SD, and minimum-maximum ranges for each of the 4 subscales and the overall scale score, which represents the mean of the 4 subscale scores. Additionally, we computed the usability percentage score, calculated as the relative value of the overall score in relation to the theoretical maximum score of the 19 items multiplied by 5 [23].

### Quantitative Treatment Satisfaction Assessment—Patient Receiving PNA-Assisted Consultations

Patient treatment satisfaction was partially assessed by the German language validated adult version of the Nutritional and Dietetic Patient Outcomes Questionnaire (NDPOQ-A) [24] completed by the patients online before and after 6 weeks and 12 weeks of PNA-assisted remote nutritional counseling. The 15-item NDPOQ-A uses a 5-point Likert scale ranging from 0 to 4 points, where higher points indicate greater agreement with statements concerning treatment satisfaction. The total questionnaire score ranges from 0 to 60. The overall mean score and SDs were calculated for all assessment time points.

Additionally, patients’ perceived impact of the PNA on health behavior was assessed after 12 weeks of PNA-assisted remote nutritional counseling using the “perceived impact” subscale of the user version of the Mobile Application Rating Scale (uMARS) [25]. This subscale consists of 6 items rated on a 5-point Likert scale, with item 5 concerning help-seeking excluded as it did not apply to this study’s scope, resulting in a total score range of 5 to 25. Mean scores, SDs, and minimum-maximum ranges were calculated for individual items and the total subscale score.

Patient-reported general self-efficacy was assessed using the validated General Self-Efficacy Scale (GSE) from Imperial College London [26] before and after 6 weeks and 12 weeks of PNA-assisted remote nutritional counseling. The GSE comprises 10 items rated on a 4-point Likert scale ranging from 1 to 4, yielding a total score range of 10 to 40, where higher scores indicate greater self-efficacy. Mean scores and SDs were calculated for all assessment time points.

### Qualitative Feedback for PNA Dashboard Evaluation

Qualitative feedback regarding the PNA dashboard was collected using a self-designed open-ended feedback questionnaire administered to both patients and dietitians online at study completion. The questionnaire assessed content quality, perceived usefulness, and visualization effectiveness of the 6 PNA dashboard panels.

### Focus Group Interviews

Two focus groups explored PNA prototype experiences: all 6 dietitians discussed tool usability and utility in semistructured interviews, while 6 patients (selected from those who provided additional written informed consent for focus group participation and were available at the scheduled interview date at the end of this trial) shared experiences with PNA-assisted remote nutritional counseling. The semistructured interview guides are presented within Multimedia Appendix 1.

All interviews (duration of 60 min each) were conducted remotely and recorded via Zoom (version 6.2.11, Zoom Video Communications, 2024) by a member of the research team (MS) and were subsequently transcribed verbatim for qualitative analysis by an independent member of the research team.

### Qualitative Analysis

Verbatim transcripts from focus group interviews and responses to open-ended feedback questionnaires were analyzed using a thematic analysis approach [27] applying a hybrid deductive-inductive methodology [28]. This qualitative strand was conducted within a pragmatic paradigm, in which the approach and methods were selected based on their utility for contextualizing and explaining quantitative outcomes rather than adherence to a single philosophical tradition consistent with the integration goals of the convergent mixed methods design [16].

The first author independently coded all transcripts using the MAXQDA software (version 24, VERBI GmbH) across 4 iterative rounds of coding. Consistent with a deductive orientation at the thematic level, an initial set of themes was defined a priori based on this study’s conceptual framework and the domains covered by the interview guides and feedback questionnaires: (1) “prior expectations and concerns,” (2) “perceived utility and satisfaction,” (3) “usability challenges,” and (4) “suggestions for clinical integration.” Within this thematic structure, codes were developed inductively from the transcript data, allowing the specific content and nuance of participant responses to shape the coding and subthemes without being constrained by predetermined categories. Subsequent coding rounds involved reviewing, refining, and consolidating codes to ensure consistency and analytical depth. Analytic decisions, including code definitions and revisions, were documented as memos within MAXQDA throughout the process.

Peer discussions to enhance the trustworthiness of the analysis were conducted in two complementary settings: a series of qualitative analysis workshop sessions involving 5 external faculty members, and multiple internal research team meetings, during which coauthors provided critical appraisal of emerging codes and subthemes and their interpretive decisions. A dedicated mapping session was subsequently conducted to organize finalized codes into subthemes within the 4 a priori themes, with the resulting code and subtheme structure reviewed and agreed upon by the coauthors.

It is acknowledged that the primary analyst (FO) is a member of the research team involved in PNA development, which may have introduced familiarity bias in the interpretation of dietitian and patient feedback. This was mitigated through the iterative coding and regular peer discussions, including members not directly involved in the tool development described above.

Quantitative and qualitative data were analyzed independently by the first author before being brought together in a convergent synthesis. Integration occurred at the interpretation stage through a narrative comparison of quantitative outcomes and qualitative theme content, with discordances and convergences between strands used to inform a more comprehensive understanding of PNA usability and implementation.

### Statistical Analysis

All primary analyses were conducted using complete cases with available data for each outcome.

Descriptive statistics for patient characteristics and lifestyle behavior include means, SD, frequencies, and percentages. For summarizing PNA-assisted consultations, means, SDs, medians, and minimum-maximum ranges are reported.

Repeated-measures ANOVA was used to assess differences in total questionnaire scores between time points (at intervention start, after 6 weeks, and after 12 weeks of PNA-assisted nutritional counseling). Paired *t* tests evaluated pre-post changes in percent weight change and BMI following PNA-assisted remote nutritional counseling.

For the descriptive report of engagement with the lifestyle-tracking app, all participants with available data were included, regardless of questionnaire completion. To ensure data quality of body weight measurements collected via the lifestyle-tracking app, recorded weights outside the range of 40‐400 kg were excluded. To address artifacts inherent to self-reported and smart-scale data (eg, synchronization errors from third-party use), subsequent body weights were also excluded if the relative change from the preceding measurement exceeded a predefined plausibility threshold. Specifically, a measurement was excluded if the proportional deviation from the previous weight exceeded 4% plus 1% per week elapsed between the two measurements. These parameters were validated by visual inspection to remove obvious outliers while accommodating natural fluctuations and rapid weight loss.

To assess the robustness of the complete-case analyses, sensitivity analyses using multiple imputation by chained equations were conducted for outcomes with participants lost to follow-up (LTFU). To explore the plausibility of the missing completely at random (MCAR) assumption, baseline characteristics between LTFU participants and completers were compared, using Wilcoxon rank-sum tests for continuous variables and Fisher exact test for binary categorical variables. For categorical variables with more than two levels, *P* values were estimated by Monte Carlo simulation (10,000 replicates). For the NDPOQ-A treatment satisfaction score and GSE self-efficacy score, missing time point values were imputed using predictive mean matching with observed scores at other time points serving as mutual predictors (mean 50 imputations, 10 iterations). As repeated-measures ANOVA cannot be directly applied to multiply-imputed data, the imputed questionnaire analyses used linear mixed models with time point as a fixed effect and a random intercept per participant, with pooled estimates derived across imputed datasets following Rubin Rules. For body weight, missing measurements ±15 days from the 12-week study end date (day 84 relative to the intervention start date) were imputed using predictive mean matching with weight values reported within the time frame of ±15 days of intervention day 0, mean mid-study weight (days 20‐65), number of weight recordings, age, BMI at treatment start, sex, and GLP-1 RA status as predictors. All imputation analyses assumed data were missing at random. Complete-case and imputed results are presented in parallel; concordance between the two approaches was used to assess the potential impact of missing data on study conclusions.

All statistical analyses were performed using R (version 4.3.0; R Foundation for Statistical Computing).

Quantitative and qualitative data were collected concurrently and analyzed separately before being integrated at the interpretation stage.

## Results

### Clinical Context and Study Population

A total of 81 adults were enrolled to receive PNA-assisted remote nutritional counseling over 12 weeks. Three participants were excluded from the analysis as they did not complete any study-related questionnaires at the intervention start, resulting in 78 participants (58/78, 74% women) included in the analytic sample. In total, 73 participants completed the primary treatment satisfaction outcome questionnaire, exceeding the prespecified minimum sample size. Loss to follow-up for individual outcomes is detailed in Figure S1 (Multimedia Appendix 1).

At enrollment, participants had a mean BMI of 32.7 (SD 4.4) kg/m^2^ and had achieved a mean weight loss of −13.3% (SD 6.7%) after 10 (SD 5) months of GLP-1 RA therapy. All participants were receiving liraglutide (Saxenda) as their GLP-1 RA regimen, with daily doses ranging from 0.6 to 3.0 mg. Key patient characteristics at the time of study enrollment are summarized in Table 1.

During the 12-week study period, participants received between 3 and 5 remote consultations. Consultation modalities included secure chat-based sessions (228/360, 63%), video calls (71/360, 20%), and phone calls (61/360, 17%). The intervention was delivered by 6 dietitians, each managing between 3 and 30 patients (median 7, IQR 11, patients per dietitian). PNA usage time per consultation ranged from 2 to 12 minutes. Dietitians shared PNA-generated outputs with participants in 50% (180/360) of consultations.

**Table 1.** Participant characteristics at enrollment.

Characteristic	Enrolled sample (N=78)
Age (years), mean (SD)	47 (11.7)
Females, n (%)	58 (74)
Body weight at treatment start (kg), mean (SD)	106.9 (17.3)
BMI at treatment start (kg/m²), mean (SD)	37.1 (4.8)
GLP-1 RA^a^ treatment duration (months), mean (SD)	9.7 (4.6)
Achieved weight loss (%), mean (SD)	13.3 (6.7)
Education, n (%)	
Higher education	22 (28)
Intermediate education	48 (62)
Basic education	4 (5)
Other	4 (5)
Comorbidities, n (%)	
Any weight-related comorbidity	68 (87)
Diabetes	3 (4)
Prediabetes	20 (26)
Dyslipidemia	56 (72)
Hypertension	31 (40)
Metabolic dysfunction-associated steatotic liver disease	11 (14)
Obstructive sleep apnea	16 (21)

a GLP-1 RA: glucagon-like peptide-1 receptor agonist.

### Engagement With the Lifestyle-Tracking App and Weight Change

During this trial’s period, participants recorded a mean of 12.7 (SD 8.5) meal and/or beverage logs per week, corresponding to an average of 30.8 (31.8) food tags weekly. The mean number of activity logs per week was 2.7 (SD 3.3), with 90% (2.4/2.7) of these entered manually. Weekly self-recorded body weight measurements average 1.2  (SD 1.0). Compared to the weeks before receiving PNA-assisted nutritional counseling, meal frequency logging decreased while the mean number of food tags per week remained stable during the intervention (Table S4 in Multimedia Appendix 1).

Body weight and BMI remained stable over the 3-month study period, with no significant changes observed (−0.2%, SD  2.5%, *P*=.56, for body weight; –0.2%, SD  3.4%, *P*=.64 for BMI). Multiple imputations of LTFU did not have an effect on the results (Table S2 in Multimedia Appendix 1). MCAR tests comparing baseline characteristics of participants with or without weight follow-up data confirmed the MCAR assumption (Table S3 in Multimedia Appendix 1). Due to supply chain disruptions affecting Saxenda availability, 21% (16/78) of participants discontinued treatment during this study’s period. Percent weight loss over the intervention period stratified for GLP-1 discontinuation or continuation is displayed in Table S3 (Multimedia Appendix 1).

### Quantitative Usability Assessment—Dietitians Delivering PNA-Assisted Consultations

The HSUS questionnaire indicated moderate overall usability for the PNA (62%, range: 45%‐67%), with higher scores for ease of use and user control, and lower scores for patient safety as well as decision and work effectiveness (Table 2). When asked whether they would recommend the PNA to a colleague, 50% (3/6) responded “yes,” and 50% (3/6) responded “no.”

**Table 2.** Dietitian-reported usability of the PNA^a^ clinical decision-support tool (HSUS^b^ questionnaire). Total mean and subscale scores range from 1 (low) to 5 (high). Min-max^c^ ranges are included for full reporting range transparency.

Subscale	Mean (SD)	Min-max
Utility: patient safety and decision effectiveness (Q1-6)	2.7 (0.5)	2.0‐3.3
Ease of use: workflow Integration (Q7-12)	3.4 (0.4)	2.8‐4
Work effectiveness (Q13-17)	2.9 (0.8)	1.6‐3.8
User control (Q18-19)	3.4 (0.6)	2.5‐4
Total mean score of all subscales	3.1 (0.5)	2.4‐3.6
Overall usability score (%)^d^	62	45‐67

a PNA: personalized nutrition advisor.

b HSUS: Healthcare Systems Usability Scale.

c Min-max: minimum-maximum.

d The overall usability score is standardized to a 10%-100% scale following the Healthcare Systems Usability Scale scoring method. Responses were provided by 6 dietitians.

### Quantitative Treatment Satisfaction Assessment—Participants Receiving PNA-Assisted Consultations

Patients reported a mean perceived impact score of 19.1 of 25 (SD 3.5) on the uMARS scale at the end of the intervention (Table 3). Patient-reported treatment satisfaction assessed by the total raw score of the NDPOQ-A (Table S6 in Multimedia Appendix 1), did not change before and after PNA-assisted nutritional counseling (41.8, SD 7.3, vs 41.2, SD 8.4, *P*=.78). Similarly, no significant change in patient-reported self-efficacy was observed on the GSE questionnaire (21.8, SD 4.5 vs 22.0, SD  4.0; *P*=.57, see Table S6 in Multimedia Appendix 1). Sensitivity analysis, including MCAR comparisons of baseline characteristics of the participants with or without LTFU of the treatment satisfaction outcome, followed by multiple imputations, showed no difference from the complete case analysis (Tables S5 and S6 in Multimedia Appendix 1). When patients were asked if they would recommend PNA-assisted remote nutritional counseling to a friend with a similar condition, 78.2% (n=61) responded “yes,” 9% (n=7) “no,” 3.8% (n=3) “I do not know,” and 7.7% (n=6) did not respond.

**Table 3.** Patient-reported perceived impact of PNA^a^-assisted consultations (uMARS^b^ questionnaire). Values are mean (SD) and minimum to maximum scores for each item range from 1 (low) to 5 (high), with a total perceived impact score ranging from 5 to 25. Responses were collected from 76 participants using the uMARS.

Question topic	Mean score (SD)	Min-max^c^
Awareness	3.9 (0.9)	2.0‐5.0
Knowledge	3.7 (0.8)	2.0‐5.0
Attitude	3.8 (0.8)	2.0‐5.0
Intention to change	3.8 (0.9)	2.0‐5.0
Behavioral change	3.9 (0.8)	2.0‐5.0
Total mean of the scale	19.1 (3.5)	10.0‐25.0

a PNA: personalized nutrition advisor.

b uMARS: user version of the Mobile App Rating Scale.

c Min-max: minimum-maximum.

### Qualitative Usability and Treatment Satisfaction Assessment

#### Overview

To gain deeper insights into the perceived utility of the PNA and treatment satisfaction with PNA-assisted consultations, we conducted qualitative assessments at the end of the intervention, including focus group interviews and open-ended feedback questionnaires. Table 4 summarizes the 4 main themes defined a priori, along with data-driven subthemes derived from the responses of both dietitians and patients.

**Table 4.** Qualitative themes and subthemes, including characterization, were based on the thematic analysis of focus group interviews and PNA^a^ feedback questionnaires among both dietitians and patients.

Theme	Subthemes	Characterization
Prior expectations and concerns	No prior expectations No concerns with receiving PNA-assisted nutritional counseling Increased weight loss motivation Improved communication with dietitian	Expectations and concerns with using the PNA or receiving PNA-supported nutritional consultations
Perceived utility and satisfaction	Brought added value to the consultations The weight prediction panel was the most appreciated and frequently used feature Weight loss empowering Not better than standard procedures Time efficiency	Overall usability of the PNA and treatment satisfaction. PNA assisted remote nutritional consultations. Specific useful features of the PNA
Usability challenges	Neglected due to output accuracy concerns or misunderstandings Limited food tag options within the app Patient tracking adherence	Nonuseful features of the PNA, including related limitations factors
Suggestions for clinical integration	Patient access streamline Automation of data transfer to the dashboard Novel panels and features Training of output interpretation and communication to patients	Requested improvements and novel ideas of the PNA. Important factors to consider when integrating the PNA to usual care

a PNA: personalized nutrition advisor.

#### Prior Expectations and Concerns

Dietitians entered this study without established expectations regarding the PNA tool’s performance or utility. However, they did harbor initial reservations concerning both accuracy and time efficiency.

*I didn't really have such great expectations because I thought, Hmm, how accurate can it be?*

[Dietitian 3]

These concerns were primarily attributed to technical implementation challenges rather than inherent limitations of the PNA system itself. The tool was hosted on a separate platform, necessitating manual entry of critical patient data, including target weights and weight surveillance timelines. Apart from these logistical challenges, they consistently reported being able to learn and effectively use the PNA tool relatively quickly, suggesting that the core functionality was intuitive and user-friendly.

*My concerns were the time required. As it is a new tool and you have to give yourself a certain amount of time to familiarise yourself and find out how to work with it. I had a lot of respect for that, because my time with the patient is already limited and if I would spend too much time with the tool, it would not have much added value for the patient. But it did not really turn out to be the case, because over time I had the feeling that you get into it pretty quickly. Many things were very self-explanatory and I did not use it to the same extent with every patient, it depended on how much time you had left and then it was discussed more or less accordingly. So, the concern was not confirmed at all*.

[Dietitian 1]

Patients expected the tool to enhance communication with their dietitians and expressed optimism about achieving improved outcomes through better discussions of treatment expectations, progress achievements, and care optimization strategies.

*I hoped that this would give me even more motivation through the communication: what is expected, what can be achieved, what can be optimised.*

[Patient 1]

#### Perceived Utility and Satisfaction

Dietitians viewed the PNA as a valuable consultation aid, strategically leveraging its features while acknowledging patient tracking limitations.

*I used the PNA as a guideline, but it didn't give me any ground-breaking new insights that I couldn't already distinguish from the [food] protocol myself; they were just a bit more precise and it helped to be able to show and explain my own assumptions supported by the PNA to the patient, for example.*

[Dietitian 2]

The weight prediction panel was the most used feature—used for both preparatory work and real-time interactions to guide patients through weight loss and maintenance.

*I especially appreciated the weight forecast feature and found it most useful during counselling sessions. It provided added value. Otherwise, I have the feeling that my guidance would be based mainly on intuition or experience, whereas the forecast offered a clearer, data-driven projection that I could share with patients.*

[Dietitian 3]

While weight trajectories generally motivated patients, some expressed discouragement during plateaus or projected gains.

*The weight forecast was extremely helpful! Motivating when it [the curve] goes down. Almost more motivating when the curve rises in the short term.*

[Patient 1]

*As my weight is stagnating, it’s demotivating.*

[Patient 2]

The current habits panel, nutritional distribution graph, and automated recommendations empowered patients by enabling actionable advice and promoting self-management.

*The current habits pane is helpful for self-monitoring and the nutritional distribution graph helps to optimise food selection. My dietitian always indicated in her feedback whether my dietary composition is OK or whether optimisations are still required.*

[Patient 4]

*The nutritionist responded with practical, real-life suggestions for how I could deal with my problems. … I really started exercising routinely 5-6 times a week and all of a sudden, I had too little protein and generally I learnt that I was eating too little protein for what I need.*

[Patient 5]

*I found the integration of nutritional assessment and exercise to be a benefit of the dashboard and useful as a starting point for counselling.*

[Dietitian 3]

Overall, patients reported increased motivation for app tracking and enhanced communication during PNA-assisted consultations.

*The more it is displayed, the greater the motivation and the more aware you are of it, which is already helping.*

[Patient 3]

*The best thing was that the dietitian took better care of you.*

[Patient 2]

#### Usability Challenges

Key usability challenges expressed by the dietitians centered around limited food tag options within the lifestyle-tracking app and the potential for inaccurate data output in patients with low tracking adherence, particularly prevalent in patients who have already achieved their weight loss goal.

*As far as eating [patterns] is concerned, I always had my doubts, because it is based on these food tags and the patients do not track so much at this stage of nutritional therapy [during weight maintenance phase] so nothing was reported back from the patients in this regard.*

[Dietitian 2]

Technical limitations further constrained the tool’s clinical utility, as the PNA model does neither provide essential information on portion sizes that would be valuable for counseling, nor does it account for changes in body composition, making interpretation more challenging when weight increases may result from lean mass gains rather than fat accumulation due to increased energy intake.

*The energy intake estimation would be helpful, but rather based on image recognition taking portion sizes into account. Otherwise, it is rather inaccurate because the patient may not tag everything.*

[Dietitian 4]

*The energy intake estimation was not really helpful, as it is not so much based on nutrition tracking but on weight development but without difference in muscle mass growth taken into account.*

[Dietitian 2]

Dietitians also reported incomplete understanding of PNA outputs, which limited the tool’s usefulness during consultations and highlighted a critical need for enhanced training in interpreting results—a requirement that dietitians themselves recognized and addressed by expressing preference for brief, 1-minute tutorials dedicated to each PNA panel that could be easily and continuously accessed for ongoing learning and reference.

*I found the [daily energy intake] graph rather confusing and difficult to interpret. During consultations, I realized it was also too complex for patients, so I didn’t use it at all.*

[Dietitian 2]

*Due to time constraints, I would have preferred brief, one-minute tutorials for each PNA panel.*

[Dietitian 6]

#### Suggestions for Clinical Integration

Despite the identified limitations, dietitians expressed willingness to continue using the PNA in daily practice, provided it achieves seamless workflow integration, and they develop a better understanding of the underlying model and its constraints. Key implementation requirements include automating patient target weight adjustments and timeline to GLP-1 RA–specific clinical weight loss milestones (defined by the Swiss Federal Office of Public Health), updates based on medical reports, eliminating manual data transfers, and enabling customizable timeframes for data visualization.

*I think it would be beneficial to integrate the weight forecast directly into our standard workflow, without the need for extra clicks or manual data transfers. I also find the integration of nutritional evaluation valuable as a useful starting point for counselling.*

[Dietitian 5]

Dietitians proposed innovative enhancements tailored to their patient population, particularly integrating daily fluid intake summaries that emphasize actual volume consumption rather than calorie content, addressing the common issue of inadequate hydration among GLP-1 RA patients who struggle to distinguish between thirst and hunger.

*I am interested in the actual volumes of unsweetened beverages, like water or tea… I have noticed that patients on GLP-1RA therapy often do not drink enough, because it can be difficult to distinguish between thirst and hunger. That is why I’d like to see fluid amounts tracked by volume, not just by calorie content…*

[Dietitian 3]

Visual improvements were strongly advocated, with suggestions to transform text-based dietary recommendations into interactive visualizations using colors, icons, and illustrations to enhance engagement and comprehension.

*Perhaps it could be presented in a more visually appealing way, using colourful and illustrative elements.*

[Patient 6]

Universal patient interest for direct PNA dashboard access was identified, with dietitians agreeing this could significantly enhance weight loss motivation, though implementation requires streamlined export processes, clear interpretation guidelines, and selective panel sharing based on data quality and individual patient needs.

*It would be highly beneficial for patients to have direct access to the PNA, particularly to support their motivation.*

[Dietitian 1]

Critical considerations include preventing patient overwhelm through appropriate information filtering, ensuring proper panel introduction during consultations, and providing dietitians with guidance on communicating potentially demotivating predictions, such as weight increases, while maintaining patient engagement and motivation throughout the treatment process.

*Although I do not count calories, the daily energy intake graph could give me a sense of my calorie intake on certain days and help guide my food choices. However, I sometimes find that too much information can be overwhelming rather than helpful…*

[Patient 7]

### Integration of Quantitative and Qualitative Findings

Integrating the quantitative and qualitative strands detected both convergence and divergence. Convergence was observed in dietitians’ usability assessments: moderate overall HSUS scores (62%), with lower ratings for patient safety, decision and work effectiveness, aligned with qualitative accounts of limited trust in lifestyle advice panels due to data completeness concerns, insufficient algorithmic transparency, and inadequate workflow integration. Both strands independently identified incomplete understanding of tool outputs as a key adoption barrier.

Divergence was identified for treatment satisfaction and self-efficacy, where quantitative measures showed no significant postintervention improvement, contrasting with qualitative reports of meaningful subjective progress, increased tracking motivation, and enhanced dietitian communication. This divergence is likely partly attributable to the limited dashboard exposure, as the PNA was shared with only 50% (39/78) of patients.

Partial convergence was observed for perceived patient impact: a mean uMARS score of 19.1/25 (SD 3.5) and 78.2% (61/78) willingness to recommend PNA-assisted counseling were broadly consistent with qualitative reports of patients valuing weight trajectory visualizations and personalized dietary feedback, though qualitative data added that motivational impact was feature-dependent and that direct dashboard access represented a universally expressed unmet need not captured quantitatively. The qualitative had reported increased motivation for app-tracking during the PNA intervention were not reflected quantitatively (Table S4 in Multimedia Appendix 1).

## Discussion

### Principal Findings

In this study, we evaluated the usability, treatment satisfaction, and weight effects of the PNA, a prototype decision-support tool designed to facilitate dietitian-led remote nutritional counseling for personalized weight management in adults achieving significant weight reduction with GLP-1 RA therapy.

Given the growing recognition that personalized nutrition is critical for patients on GLP-1 RA therapy to maximize health outcomes, prevent nutritional deficiencies, manage side effects, and support sustainable weight management [9], tools such as the PNA could help address important needs in this area, such as providing quantitative and qualitative advice based on app-based lifestyle tracking.

Overall, PNA-assisted counseling was feasibly delivered within routine care, and patients maintained clinically significant weight loss over the 12-week intervention. The mixed methods design of this study allowed for a more nuanced interpretation of these findings than quantitative measures alone would permit. Notably, with respect to the patient satisfaction and self-efficacy findings, a discordance was identified between quantitative null findings and positive qualitative signals, which is a well-recognized phenomenon in mixed methods usability studies of novel health technologies [29,30]. This underscores the limitation of applying conventional efficacy metrics to prototype evaluations and illustrates the added value of a convergent parallel mixed methods approach in capturing the full complexity of technology implementation. The limited PNA dashboard exposure to 50% (39/78) of patients likely attenuated measurable effects, and the universal patient desire for direct dashboard access represents a clear priority for future development despite being designed as a clinical decision support tool for dietitians as primary users.

Moreover, our qualitative findings recognized pronounced feature-specific variations in dietitians’ assessment of the PNA’s utility, highlighting the need for targeted improvements across different tool components. The weight prediction panel was identified as the most valued and frequently used feature, with dietitians successfully integrating it into both consultation preparation and real-time patient interactions to support both weight loss and maintenance phases. This feature’s success appears driven by its primary dependence on continuous weight tracking—data that patients found comparatively low-burden to record consistently (1/wk)—combined with its ability to provide concrete, motivational visualizations that patients could readily understand and engage with. While the prediction model also incorporates food and activity data to refine trajectory estimates, its robustness to incomplete dietary logging meant that clinically meaningful predictions remained accessible even when food tracking adherence was suboptimal. In contrast, panels whose outputs were more directly dependent on complete food and activity tracking data encountered greater skepticism with dietitians citing incomplete data inputs, limited food tag options, insufficient algorithmic transparency, and the model’s inability to account for portion sizes or body composition changes were identified as key concerns undermining their trust.

Technical implementation barriers, including the prototype’s separate hosting platform, manual data entry requirements (of target weights and weight surveillance timelines), and the incomplete understanding of the PNA outputs, particularly regarding the underlying algorithms and appropriate interpretation of predictions, further constrained adoption and limited the perceived clinical utility. The latter knowledge gap may have prevented effective communication of results to patients and reduced confidence in tool recommendations, creating a barrier to adoption even when data quality was adequate.

These findings align with established frameworks for digital health adoption barriers, which identify technical, organizational, and user-level barriers as key determinants of implementation success [29-31]. The PNA’s integration into clinical practice will require iterative codevelopment with dietitians and patients, seamless embedding within existing digital platforms, and structured onboarding that builds algorithmic literacy and clinical confidence in tool outputs. Previous research has shown that apps and platforms developed in collaboration with dietitians are more trusted and accepted by both professionals and patients [32,33]. Our observations also highlight the need for sufficient time for training and adaptation when adopting new technology, including the provision of continuous training materials [30,34].

App engagement, including regular tracking of lifestyle behaviors and body weight, is essential not only for accurate weight prediction [5,35] but also for optimizing outcomes during GLP-1 RA–based weight management therapy, as recent evidence demonstrates that greater engagement with digital platforms significantly enhances weight loss [36]. The NICE (National Institute for Health and Care Excellence) in the United Kingdom also recognizes the important role of digital tools in weight management and provided guidance on their integration into clinical practice [37]. According to this guidance, digital aids should promote weight goal achievement through ongoing engagement, personalized feedback, and tailored advice. While the PNA was designed to meet these requirements, bringing it into daily use will require further refinement and robust efficacy evaluation in its final form.

Several technical enhancements could substantially increase the PNA tools’ utility. Technical improvements, such as the use of computer vision and depth-sensing technologies for quantitative nutrient analysis, enabling 3D reconstruction of food portions (rather than relying on standard serving size assumptions) [38], as well as mobile voice-added apps for food intake reporting [39], are promising. Additionally, large language models offer new opportunities to refine nutritional recommendations based on individual profiles (eg, recipe suggestions tailored to specific requirements) [40,41]; however, optimization of nutritional composition estimations is still required [42]. Further development should include options for direct patient interaction with the tool and prompts emphasizing that consistent lifestyle tracking improves the quality and accuracy of support provided. In the future, our energy balance model could incorporate individual body composition data from connected smart scales, moving beyond generic assumptions and further enhancing the tool’s utility for optimizing both body weight and composition—key factors for long-term health and quality of life. Additionally, integrating self-reported GLP-1 RA dosages tracked within the lifestyle app into the PNA dashboard could support personalized dose adjustments based on predicted trends. Manufacturer-linked companions such as WeGoTogether (Novo Nordisk Inc) and Lilly Health (Eli Lilly and Company) offer comparable functionality, but remain unavailable outside of the United States, leaving a clear gap that the PNA could cover.

In the context of the nutritional priorities associated with GLP-1 RA therapy, several areas for further development of tools such as the PNA can be identified. To address the risk of micronutrient deficiencies under energy restriction, future iterations should incorporate monitoring of micronutrients such as B vitamins, calcium, vitamin D, iron, folate, and trace minerals such as zinc [9,12-15]. Specific dietary adjustments can further meaningfully reduce GLP-1 RA–related gastrointestinal side effects, especially during initiation and dose escalation [9]. Psychological factors such as disordered eating [43], depression [44], and quality of life [45] represent further dimensions that the PNA could eventually incorporate. Finally, just-in-time interventions using behavioral change techniques, triggered by detection of dietary lapses or deviations from target weight trajectories [46,47], also represent a promising avenue for future development.

Several limitations in this study’s design should be acknowledged. The single-arm trial design limits the ability to assess clinical efficacy; while our primary focus was on usability and treatment satisfaction, future health technology efficacy assessments of the finalized product should be conducted in randomized controlled trials. The dietitians involved (predominantly middle-aged women) may not represent the broader professional community, particularly younger practitioners who may be more receptive to digital tools. The requirement for a minimum of 16 weeks of prior GLP-1 RA therapy in combination with lifestyle-app tracking before enrollment may limit the generalizability of findings to individuals earlier in their treatment trajectory or those with limited prior engagement with lifestyle tracking, as participants had by definition already passed the initial weight loss phase and accumulated sufficient data to inform the PNA tool. The patient focus group sample may introduce a positive selection bias, as participation required additional written informed consent and evening availability, potentially overrepresenting more motivated patients. However, as open-ended feedback questionnaires, which formed an equally important qualitative data source, were completed by 76 of 78 patients, the overall qualitative findings are unlikely to be substantially affected by this sampling limitation. Finally, supply chain issues with GLP-1 RA led to abrupt drug discontinuation in at least 21% (16/78) of the participants, complicating the interpretation of intervention effects on weight. Although abrupt pharmacotherapy withdrawal is known to predispose to weight regain [10,11], this was not observed in our cohort. However, the absence of expected weight regain following pharmacotherapy discontinuation in our cohort cannot be definitely attributed to PNA-assisted nutritional counseling due to the lack of a control group.

### Conclusions

Clinical decision support systems that integrate digital lifestyle tracking and advanced analytics may help address nutritional priorities during GLP-1 RA weight management. This could support scalable and adaptive dietitian-led nutrition care, given their limited time and capacity to analyze large, multidimensional data streams. Further development, rigorous user-centered design, and robust clinical efficacy testing are necessary to fully realize the potential of such systems to improve health outcomes.

## Supplementary material

10.2196/81112 Multimedia Appendix 1 Supporting figures, tables, and interview guides.

## Abbreviations

GLP-1: glucagon-like peptide-1
GLP-1 RA: glucagon-like peptide-1 receptor agonist
GSE: General Self-Efficacy Scale
HSUS: Healthcare Systems Usability Scale
LTFU: lost to follow-up
MCAR: missing completely at random
NDPOQ-A: adult version of the Nutritional and Dietetic Patient Outcomes Questionnaire
NICE: National Institute for Health and Care Excellence
PNA: personalized nutrition advisor
uMARS: user version of the Mobile Application Rating Scale
