# Supplementary material for: Clinical Decision Support for Digital Dietary Counseling Under GLP-1 Receptor Agonist Therapy: Convergent Mixed Methods Usability and Treatment Satisfaction Pilot Study of the Personalized Nutrition Advisor
Source: JMIR Form Res. 2026 Jul 21;10:e81112. doi: 10.2196/81112 (PMC13423961; doi:10.2196/81112)
Supplement: Multimedia Appendix 1 [file formative-v10-e81112-s001.docx]

**
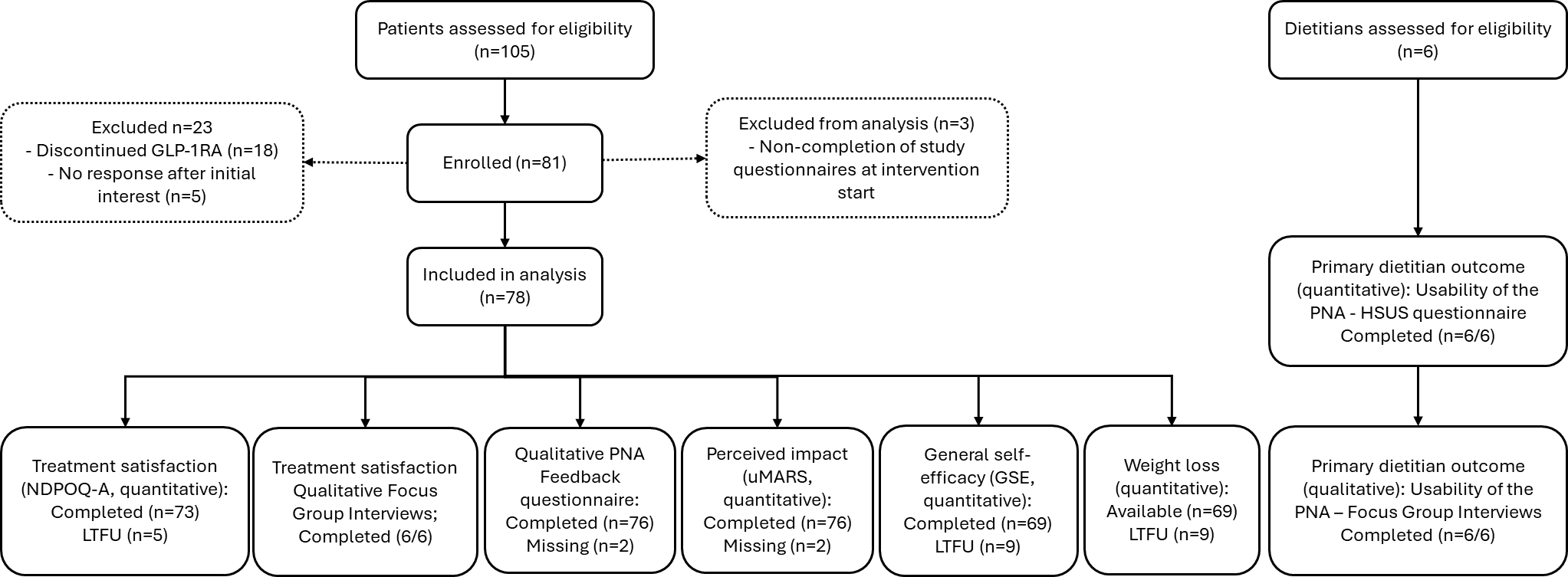
**

**Figure S1.** Participant flowchart including an overview of number of participants screened, enrolled, non-eligible, lost to follow-up, reasons for exclusion and details on data missingness for each outcome.

**Table S1.** Daily thresholds-based triggers for a complete diet plan (derived from Swiss nutritional guidelines by the Federal Food Safety and Veterinary Office, FSVO [1]) and generic recommendations in case of threshold violation for the “action” column of the “Nutritional Advice” panel of the PNA dashboard.

| **Nutrient** | **Daily thresholds** | **Generic recommendations in case of threshold(s) violation(s)** |
| --- | --- | --- |
| **Protein** | A minimum of 0.8 g/kg/day of ideal body weight (= body weight at BMI 25 kg/m²), ideally 1.2–1.6 g/kg/day | Increase intake of protein rich foods like lean meat, fish, dairy products, eggs and legumes. |
| **Added sugar** | Max. 5 % of total energy intake/day | Avoid products with added sugar like sweet yoghurts, sweets, cakes, cookies and sweet beverages. |
| **Saturated fatty acids (SFA)** | Max. 10 % of total energy intake/day | Decrease consumption of animal source foods like meat and meat products, butter and dairy products. |
| **Proxy for dietary phytochemical exposure** | DPI score > 28.8 (Dietary phytochemical index = daily energy derived from phytochemical-rich foods (kcal) / total daily energy intake (kcal) × 100) [2] | Increase intake of phytochemicals by eating more vegetables, fruits and legumes. |
| **Fibre** | Min. of 14.6 g/1000 kcal | Increase intake of whole grain products, vegetables, fruit, nuts, seeds and legumes. |

**Table S2.** Percent weight loss during the 12 weeks of PNA-assisted care stratified for GLP-1RA usage throughout the trial period.

| Analysis | GLP-1 group | N | % Weight change | |
| --- | --- | --- | --- | --- |
|  |  |  | **Mean (SD)** | **p-value** |
| **Complete-case** | **All patients** | **69** | -0.18 (2.52) | 0.556 |
|  | Discontinued GLP-1 | 16 | 0.8 (2.6) | 0.237 |
|  | Continued GLP-1  (at lower dose) | 39 | -0.68 (2.53) | 0.1 |
|  | Unknown / Not recorded | 14 | 0.1 (2.16) | 0.869 |
| **Multiple imputation (m = 50)** | **All patients** | **75** | -0.14 (2.47) | 0.616 |
|  | Discontinued GLP-1 | 18 | 0.87 (2.47) | 0.153 |
|  | Continued GLP-1  (at lower dose) | 42 | -0.66 (2.48) | 0.092 |
|  | Unknown / Not recorded | 15 | 0.1 (2.16) | 0.869 |

**Table S3.** Participant characteristics stratified by weight follow-up data availability.

|  | **Mean (SD) or n/N (%)** | |  |
| --- | --- | --- | --- |
| **Characteristic** | **LTFU (n = 9)** | **Completers (n = 69)** | **p-value** |
| Age (years) | 42.2 (10.5) | 47.6 (11.7) | 0.152 |
| No. of females | 6/9 (66.7%) | 52/69 (75.4%) | 0.687 |
| Body weight at treatment start (kg) | 107.3 (13.2) | 106.9 (17.8) | 0.772 |
| BMI at treatment start (kg/m²) | 37.1 (2.6) | 37.1 (5) | 0.584 |
| GLP-1RA treatment duration (months) | 10.7 (4.7) | 9.6 (4.6) | 0.448 |
| Achieved weight loss (%) | 11.4 (7.9) | 13.6 (6.5) | 0.628 |
| **Education** |  |  |  |
| Higher education | 4/9 (44.4%) | 18/69 (26.1%) | 0.261 |
| Intermediate education | 5/9 (55.6%) | 43/69 (62.3%) | 0.727 |
| Basic education | 0/9 (0%) | 4/69 (5.8%) | 1 |
| Other | 0/9 (0%) | 4/69 (5.8%) | 1 |
| **Comorbidities** |  |  |  |
| Any comorbidity | 7/9 (77.8%) | 61/69 (88.4%) | 0.324 |
| Diabetes | 0/9 (0%) | 3/69 (4.3%) | 1 |
| Prediabetes | 2/9 (22.2%) | 18/69 (26.1%) | 1 |
| Dyslipidemia | 7/9 (77.8%) | 49/69 (71%) | 1 |
| Hypertension | 2/9 (22.2%) | 29/69 (42%) | 0.304 |
| Fatty liver disease | 1/9 (11.1%) | 10/69 (14.5%) | 1 |
| Obstructive sleep apnea | 1/9 (11.1%) | 15/69 (21.7%) | 0.676 |

Mean (SD) reported for continuous variables; n (%) for categorical variables. Weight follow-up defined as at least one body weight measurement within ±15 days of both day 0 (intervention start) and day 84 (end of study). LTFU = lost to follow-up. Continuous variables: Wilcoxon rank-sum test. Categorical binary variables: Fisher’s exact test and categorical multi-level variables with Monte Carlo simulation.

**Table S4**. Tracking frequency per week by modality before and after the 12-week period with PNA-assisted remote nutritional counselling. Mean and SD were calculated around the start/end of intervention date +15 days.

| **App tracked modality** | **Before intervention Mean (SD)** | **After 12-week intervention Mean (SD)** | **p-value** |
| --- | --- | --- | --- |
| No. of meal and beverage logs/week | 7.9 (4.6) n=75 | 7.0 (4.8) n=66 | 0.006 |
| No. of food tags/week | 17.6 (16.7) n=75 | 18 (19.2) n=66 | 0.762 |
| No. of activity logs/week | 1.7 (1.85) n=36 | 1.94 (1.86) n=32 | 0.877 |
| No. of body weight logs/week | 0.8 (0.7) n=70 | 0.8 (0.6) n=60 | 0.411 |
| Mean (SD) of weekly logs per participant. LTFU= loss to follow up. Before intervention: mean of ±15 days around day 0 (PNA start). After intervention: mean of ±15 days around day 84 (end of study). p-value: paired t-test comparing before vs after intervention. n=number of patients with data points. Only participants with data in both windows are included in p-value calculation. | | | |

**Table S5.** Participant characteristics stratified by treatment satisfaction (NDPOQ-A) follow-up data availability**.**

|  | **Mean (SD) or n/N (%)** | |  |
| --- | --- | --- | --- |
| **Characteristic** | **LTFU (n = 5)** | **Completers (n = 73)** | **p-value** |
| Age (years) | 41.6 (10.8) | 47.4 (11.7) | 0.266 |
| No. of females | 3/5 (60%) | 55/73 (75.3%) | 0.598 |
| Body weight at treatment start (kg) | 116.9 (23.8) | 106.2 (16.7) | 0.185 |
| BMI at treatment start (kg/m²) | 38.9 (5.2) | 37 (4.7) | 0.338 |
| GLP-1RA treatment duration (months) | 9.1 (4.7) | 9.8 (4.6) | 0.775 |
| Achieved weight loss (%) | 7.9 (5.3) | 13.7 (6.6) | 0.061 |
| **Education** |  |  |  |
| Higher education | 1/5 (20%) | 21/73 (28.8%) | 1 |
| Intermediate education | 4/5 (80%) | 44/73 (60.3%) | 0.644 |
| Basic education | 0/5 (0%) | 4/73 (5.5%) | 1 |
| Other | 0/5 (0%) | 4/73 (5.5%) | 1 |
| **Comorbidities** |  |  |  |
| Any weight-related comorbidity | 4/5 (80%) | 64/73 (87.7%) | 0.506 |
| Diabetes | 0/5 (0%) | 3/73 (4.1%) | 1 |
| Prediabetes | 1/5 (20%) | 19/73 (26%) | 1 |
| Dyslipidemia | 4/5 (80%) | 52/73 (71.2%) | 1 |
| Hypertension | 1/5 (20%) | 30/73 (41.1%) | 0.643 |
| Metabolic dysfunction-associated steatotic liver disease | 1/5 (20%) | 10/73 (13.7%) | 0.543 |
| Obstructive sleep apnea | 1/5 (20%) | 15/73 (20.5%) | 1 |

Mean (SD) for continuous variables; n (%) for categorical variables. LTFU = lost to follow-up for the primary outcome. Continuous variables: Wilcoxon rank-sum test. Categorical binary variables: Fisher’s exact test and categorical multi-level variables with Monte Carlo simulation.

**Table S6.** Patient reported treatment satisfaction (NDPOQ-A) and self-efficacy (GES) before and after 6 and 12 weeks of PNA assisted remote nutritional counselling.

| **Questionnaire and timepoint** | **Complete-case analysis**  **n** | **Mean (SD)** | **Multiple imputation (m = 50)**  **n** | **Mean (SD)** |
| --- | --- | --- | --- | --- |
| **Treatment satisfaction (NDPOQ-A)** |  |  |  |  |
| Intervention start | 73 | 41.78 (7.26) | 78 | 41.94 (7.44) |
| 6 Weeks | 73 | 41.47 (7.49) | 78 | 41.65 (7.63) |
| 12 Weeks | 73 | 41.19 (8.36) | 78 | 41.47 (8.38) |
| **General Self-efficacy (GSE)** |  |  |  |  |
| Intervention start | 69 | 21.78 (4.48) | 75 | 21.65 (4.53) |
| 6 Weeks | 69 | 21.55 (4.17) | 75 | 21.48 (4.21) |
| 12 Weeks | 69 | 22.04 (3.91) | 75 | 22.03 (4) |

This table presents the mean total raw scores and SD of the standardised NDPOQ-A (overall score range: 0–60 points) and GSE (overall score range: 6–30) questionnaires, collected at intervention start, after 6 weeks, and after 12 weeks of PNA-assisted nutritional counselling. Higher scores reflect greater satisfaction and self-efficacy. n indicates the number of participants included for the complete case calculation of the group mean and the multiple imputations for each respective questionnaire at each time point. Observed scores across timepoints served as mutual predictors. Pooled estimates follow Rubin’s rules. Participants lacking complete questionnaires at intervention start where excluded from the analysis.

## **Semi-Structured Patient Interview Guide:**

1. When comparing the past three months of PNA supported nutritional counselling to the standard counselling you have had before, which treatment method gave you the most understandable nutritional advice?
2. Why?
3. What were your expectations when consenting to the study?
4. What were your concerned when consenting to the study?
5. How have your expectations changed over the past three months?
6. How have your concerns changed over the past three months?
7. What did you like the most with the PNA supported nutritional counselling?
8. What did you dislike the most with the PNA supported nutritional counselling?
9. What are your general impressions of receiving PNA supported nutritional counselling?
10. What are your suggestions for improvement of the PNA for future use?

## **Semi-Structured Dietitian Interview Guide:**

1. What expectations did you have when you agreed to participate in the study?
2. Have your expectations changed over the past few months?
3. What concerns did you have when you agreed to participate in the study?
4. How have those concerns changed in recent months?
5. What did you like most and least about the PNA-supported nutritional counselling?
6. If you focus exclusively on the PNA dashboard (without considering the additional study-related processes) and compare it with standard tools, which method allowed you to get an overview of your patients' status more efficiently?
7. If the PNA were integrated into the usual care workflow (without an additional platform and connection, e.g. so that body weight goals would be automatically adjusted), would you still prefer the conventional method over the PNA?
8. How could the PNA be presented to all nutritionists in a simple and understandable way to avoid confusion?

The PNA is designed as a support tool for nutritionists like you and is not intended to replace your work. What do you feel is still missing from this tool or could be improved? (Please do not focus on limitations in app interaction or suboptimal food image recognition.)

**References**

1. Federal Food Safety and Veterinary Office, FSVO. Swiss Dietary Recommendations. Accessed April 19, 2026. <https://www.blv.admin.ch/blv/en/home/lebensmittel-und-ernaehrung/ernaehrung/empfehlungen-informationen/schweizer-ernaehrungsempfehlungen.html>
2. Hamedi-Shahraki S, Jowshan MR, Zolghadrpour MA, Amirkhizi F, Asghari S. Dietary phytochemical index is favorably associated with oxidative stress status and cardiovascular risk factors in adults with obesity. *Sci Rep.* 2023;13:7035. doi:10.1038/s41598-023-34064-4
